# Overweight and Obesity among Sexual Minority Adults in the United States

**DOI:** 10.3390/ijerph16101828

**Published:** 2019-05-23

**Authors:** Sunday Azagba, Lingpeng Shan, Keely Latham

**Affiliations:** Department of Family and Preventive Medicine, University of Utah School of Medicine, Salt Lake City, UT 84108, USA; lingpeng.shan@utah.edu (L.S.); keely.latham@utah.edu (K.L.)

**Keywords:** overweight, obesity, sexual minority populations, lesbian, gay, bisexual

## Abstract

There is evidence that sexual minority populations have a potentially heightened risk of poor health outcomes due in part to the discrimination they may face. In the present study, we examined whether overweightness and obesity vary by sexual minority subgroup using a large, nationally representative sample. Data were drawn from 2014–2017 Behavioral Risk Factor Surveillance System (BRFSS) surveys (*n* = 716,609). We grouped participants according to sexual identity (straight, lesbian or gay, bisexual, and other/don’t know/not sure). The propensity score matching technique was used to address covariate imbalance among sexual identity groups. In addition, subgroup analyses were performed for both males and females. Compared to straight adults, lesbian females had significantly higher odds of being overweight (OR (odds ratio) 1.33; 95% CI (confidence interval) 1.17–1.53), whereas gay males had significantly lower odds (OR 0.66; 95% CI 0.59–0.73). Similarly, lesbians were more likely to be obese (OR 1.49; 95% CI 1.31–1.70), whereas gay men had significantly lower odds of obesity (OR 0.77; 95% CI 0.69–0.86) when compared to straight adults. Bisexual females had significantly higher odds of being overweight (OR 1.21; 95% CI 1.10–1.34) and obese (OR 1.43; 95% CI 1.29–1.59), whereas bisexual males showed no significant difference. Our results strengthen previous findings and further highlight the need for research by sexual minority subgroup.

## 1. Introduction

Overweightness and obesity remain significant public health issues in the United States. In 2015, nearly 40% of all U.S. adults were obese (body mass index or BMI ≥ 30) [1]. Obesity is associated with significant health risks, including hypertension, type 2 diabetes, coronary heart disease, stroke, and more [2,3]. Accumulating evidence points to a positive association between obesity and some types of cancers. When compared to women with a healthy weight, overweight and obese women have significantly higher odds of developing endometrial cancer; obese women with a BMI ≥ 40 are seven times as likely to develop this cancer [4]. Obese individuals also have increased risk of several other cancers including liver [5], pancreatic [6], colorectal [7] and other cancers [8,9,10,11,12].

There is evidence that certain populations have a potentially heightened risk of overweightness and obesity (e.g., those with low socioeconomic status, older adults, and those with certain medical problems [13]). Some prior studies suggest that sexual minority populations, such as lesbian, gay, and bisexual (LGB) groups, are more likely to be overweight when compared to straight peers. In addition, previous research has found that when compared to heterosexual counterparts, lesbian and bisexual females have a higher risk of overweightness and obesity [14], whereas gay males have a decreased risk [15,16]. There are limited population-based studies in the existing literature on LGB and body weight status, with much of what is currently known studied in small or regional samples and/or convenience sampling [16,17,18,19].

The high prevalence of overweightness and obesity among certain sexual minority populations is thought to be related to prejudice, homophobia, and amplified levels of stress that sexual and gender minorities face [20]. The minority stress model posits that stigma and discrimination contribute to heightened stress levels among sexual minority populations [20]. In addition, prior research suggests that the various health disparities observed among sexual minority populations, such as the high prevalence of past substance use [21], depression [22,23], and anxiety [22,23], may arise from stressors induced by hostility, discrimination, and victimization [20]. The minority stress framework may provide important information that is valuable for policy decisions aimed at addressing health disparities among sexual minorities [24]. Stress has also been separately associated with obesity. Cortisol, the primary stress hormone, was found to be higher in obese individuals [25]. Stress-eating may be a way for individuals to cope with stress [26,27].

The Institute of Medicine and the National Institutes of Health have indicated the need for quality research to provide a better understanding of the health of sexual minorities [28,29]. In the present study, we examined whether overweightness and obesity vary by sexual minority subgroup using a large, nationally representative sample and a rigorous analytic approach. Identifying a subpopulation with heightened risks of engaging in negative health behaviors is particularly important for prevention targeting.

## 2. Materials and Methods

### 2.1. Data

We combined data from the 2014–2017 Behavioral Risk Factor Surveillance System (BRFSS) surveys. BRFSS was designed in the early 1980s by the Centers for Disease and Prevention (CDC) to collect data on noninstitutionalized residents’ (age ≥ 18) health-related risk behaviors and events, chronic health conditions, and use of preventive services. The BRFSS uses two steps for weighting purposes: design weighting and iterative proportional fitting, also known as raking [30]. Since 2011, the BRFSS has collected participant information from all 50 states, the District of Columbia, and three U.S. territories using disproportionate stratified sampling for landlines and random sampling for cellular telephones [30]. With over 400,000 adult interviews completed annually, it is the largest, consistently conducted health-related survey in the world. The survey measures have proven to be both reliable and valid [31]. The BRFSS survey contains a core component, optional modules, and state-added questions. Questions related to sexual orientation are included in the sexual orientation and gender identity module. The module is optional, and the number of participating states varies from year to year (19 in 2014, 21 in 2015, 25 in 2016, and 27 in 2017). We restricted our study sample to the 716,609 responders who answered the question(s) related to sexual orientation in the Sexual Orientation and Gender Identity module in the combined 2014–2017 BRFSS data.

### 2.2. Measures

#### 2.2.1. Dependent Variables

Our outcomes of interest were overweightness and obesity. Following CDC standards, we created two dichotomous variables using BMI to define overweight (BMI ≥ 25 kg/m^2^) and obese (BMI ≥ 30 kg/m^2^). BMI is a tool to measure body fat and is calculated by dividing a person’s weight in kilograms by the square of their height in meters [32].

#### 2.2.2. Independent Variables

Sexual identity was assessed by respondents’ selection of one of the following responses to “Do you consider yourself to be”: “Straight,” “Lesbian or gay,” “Bisexual,” “Other,” “Don’t know/Not Sure,” “Refused,” and “Not asked or Missing”. In our study, we excluded respondents who refused or did not answer the question and combined respondents who answered “Other” and “Don’t know/Not Sure” into one category. In terms of the other covariates, age was categorized as 18–24, 25–34, 35–44, 45–54, 55–64, and 65+. Race was grouped as White, Black, Hispanic, and other. Sex, marital status, education level, employment, and income were obtained from related survey questions. We further classified employment as yes (employed for wages or self-employed) or no. We used the respondent’s state identification to classify our subjects into 1 of 4 regions using the same regional classification system as the U.S. Census Bureau [33]; we classified Guam as part of the Western region.

### 2.3. Statistical Analysis

Demographic and socioeconomic characteristics were described by the sexual identity groups (straight, lesbian or gay, bisexual, other/don’t know/not sure). The weighted frequency was reported for all categorical variables. Rao-Scott chi-square tests were used for comparing characteristics among the four groups. To address the potential imbalance in the distribution of observed characteristics between subgroups, we used a propensity score model (PSM) [34,35]. The PSM potentially minimizes selection bias in observational studies by ensuring that the comparability of study groups on observed characteristics are as similar as possible. For the PSM analysis, we used a flexible approach, generalized boosted model (GBM), to estimate propensity score weights for multiple treatments [36,37].

Recent advances in PSM have shown that machine learning methods (e.g., GBM) perform better than parametric approaches (e.g., logistic regression for binary case) in terms of bias reduction and mean squared error [36,37]. We used the algorithm developed by McCaffrey et al. [36] for multinomial propensity scores function in the Toolkit for Weighting and Analysis of Nonequivalent Groups (twang) package [36,38]. Twang package uses GBM in estimating propensity score weights. It relies on an iterative process of fitting regression trees, whereby at each subsequent iteration a new tree is selected to provide the best fit to the residuals of the model from the prior iteration [38]. Responders’ characteristics including age, sex, race, education level, employment status, and region were incorporated into the model estimating propensity score weights. To account for the complex survey design, we used the sampling weights in the PSM analysis, and used the sampling weight multiplied by the propensity score weight as the final propensity score weight in the outcome analyses. As shown in prior simulation studies, this strategy allowed us to produce better population-level treatment effect estimates [38,39]. We assessed a key assumption required for valid estimates to be obtained from PSM, an overlap assumption, which states that each individual has a positive probability of receiving each treatment. Boxplots were used to compare the distribution of propensity scores across groups in order to assess the overlap assumption. In addition, an imbalance test was performed to check whether there was a balance between groups by comparing absolute standardized mean differences (ASMD) between the treatment groups on the observed characteristics, before and after weighting. After weighting, the ASMD should significantly decrease if sufficient balance is achieved [37].

In the outcome model, we examined the association between sexual identity and overweightness/obesity after deriving the weights from the PSM analysis. Each outcome was regressed on a categorical variable representing sexual identity status with heterosexual being the reference group. Separate subgroup analyses were performed for male and female adults. The analysis used the derived combined weight in the outcome analysis (sampling weight multiplied by the propensity score weight) [38,39]. All of the statistical analyses were performed using SAS 9.4 (SAS Institute, Inc., Cary, NC, USA).

## 3. Results

Table 1 describes the demographic and socioeconomic characteristics of the study cohort by sexual identity. Of all 716,609 responders, 95.6% were straight, 1.4% were lesbian or gay, 1.6% were bisexual, and the remaining 1.4% were other/don’t know/not sure. Compared to straight adults, demographic and socioeconomic characteristics of sexual minorities were significantly different. Straight adults were more likely to be aged 55 years and older (68.6%), and sexual minorities were more likely to be aged 34 and younger (39.9% for lesbian or gay and 61.5% for bisexual). Gay adults were more likely to be male (60.9%). Other/don’t know/not sure had the highest percentage of Hispanic people (36.0%). Other/don’t know/not sure also had the highest percentage of people that did not graduate from high school (36.1%) and unemployed people (54.7%).

**Figure 1:**
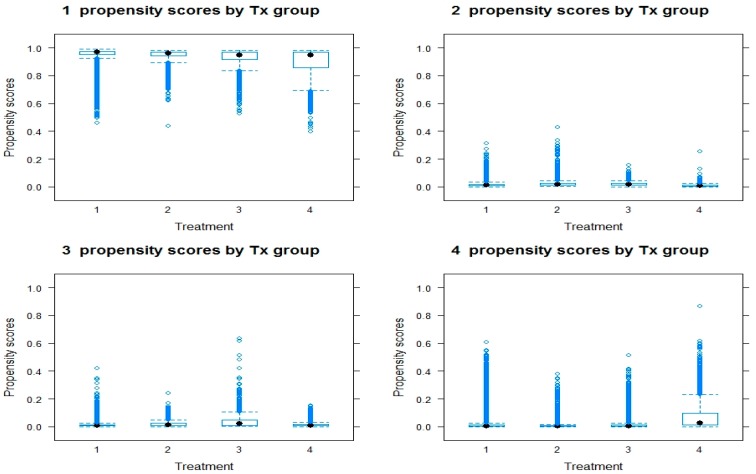
Box plots of propensity scores distribution. 1 = Straight; 2 = Gay/Lesbian; 3 = Bisexual; 4 = Other/Don’t know/ Not Sure.

Table 2 presents results from the PSM diagnostic checks including the ASMD before and after weighting. All ASMD decreased under 0.20 after PSM, which indicates sufficient balance was achieved. Boxplots show the distribution of propensity scores across groups (Figure 1) indicating that the overlap condition was met. Table 3 presents the result of the PSM analysis of overweightness (BMI ≥ 25) on sexual identity status stratified by sex. Results showed a significant association between sexual identity and bodyweight status. Specifically, bisexual adults were more likely to be overweight (OR 1.08; 95% CI 1.00–1.17) when compared to straight adults. Adults with other/don’t know/not sure sexual identity were less likely to be overweight (OR 0.83; 95% CI 0.73–0.94). Among male adults, gay (OR 0.66; 95% CI 0.59–0.73) and other/don’t know/not sure adults (OR 0.76; 95% CI 0.62–0.94) were less likely to be overweight. Lesbian (OR 1.33; 95% CI 1.17–1.53) and bisexual (OR 1.21; 95% CI 1.10–1.34) females were more likely to be overweight compared to their straight peers.

Table 4 reports the result of the PSM analysis of obesity (BMI ≥ 30) on sexual identity status stratified by sex. Significantly higher odds of obesity were found among bisexual adults (OR 1.29; 95% CI 1.18–1.42) when compared to straight adults. Lower odds of obesity were found among the other/don’t know/not sure group (OR 0.88; 95% CI 0.78–1.00) compared to straight adults. When comparing sexual minority females to straight females, we found that lesbian (OR 1.49; 95% CI 1.31–1.70) and bisexual (OR 1.43; 95% CI 1.29–1.59) females had higher odds of being obese. Gay males had lower odds of being obese than their straight counterparts (OR 0.77; 95% CI 0.69–0.86).

## 4. Discussion

Many previous studies have found that LGB populations may be disproportionally impacted by overweightness and obesity. However, findings are mixed, as other studies have found no significant difference between the weight of certain sexual minority subgroups and their heterosexual counterparts [16]. Overweightness and obesity remains a significant public health issue, and related outcomes should be studied by sexual minority subgroup in order to avoid masking important differences.

We found that in the overweight group, the full sample of lesbian and gay individuals had lower odds of overweight (although not significant). However, when analyzed by sex, females had significantly higher odds of being overweight, whereas males had significantly lower odds. This is consistent with past research findings that lesbian females had significantly higher rates of being overweight [17] and males were less likely to be overweight [16] when compared to heterosexual counterparts. This highlights the need for an examination of sexual minority populations by subgroups in order to avoid masking important differences. Grouping lesbian and gay groups together results in misleading findings, as the lower odds appear driven by gay males. Similarly, grouping together bisexuals of both sexes may also provide unreliable results. In the full sample, it appeared that all bisexuals had significantly higher odds of being overweight. In a sex-based stratified analysis, it was revealed that female bisexuals still had significantly higher odds of being overweight, which is consistent with prior research [40]. However, male bisexuals had non-significant lower odds of being overweight.

The importance of examining sexual minority subgroups is also evident in the analysis of obese individuals. While the full sample of lesbian and gay adults does not display significant odds related to obesity, a sex-based stratified analysis revealed important differences. Gay males had lower odds of obesity, and lesbian females had higher odds of obesity. This is consistent with past research conducted in small or regional samples that found lesbian females had higher rates of obesity [17] and that gay males had lower rates of obesity when compared to heterosexual counterparts [16,19]. In the bisexual group, obesity results also differed by sex. The full sample found that bisexuals had higher odds of obesity compared to straight individuals, although once separated by sex, only bisexual females had significantly higher odds of obesity.

The MST explains that sexual minorities experience unique stigmas and discrimination that can lead to poorer health outcomes [20]. Stress-eating may be a way for individuals to cope with stress [26,27]. The association between sexual minority stress and unhealthy weight has been studied in a variety of contexts, including weight-gain-trajectory [41], binge eating disorder [42,43], and other eating disorders [44]. Studies have found that gay, bisexual, or unsure men have higher rates of eating disorders and disordered eating behaviors compared to heterosexual men [44]. This partly coincides with our findings that gay men have significantly lower odds of overweightness or obesity, and that males identifying as “Other/Don’t know/Not sure” have lower odds of being overweight. It is unclear to what extent the minority stress model can help explain our findings that lesbian and bisexual females had higher odds of being overweight and obese compared to heterosexual peers.

A possible explanation for the increased odds of overweightness and obesity found in lesbian and bisexual females remains a complex issue. Some have criticized the labelling of lesbian people as an at-risk population for overweightness and obesity, highlighting that many believe that ‘lesbian culture’ is to blame [45]. We speculate that a variety of factors contribute to the heightened risk of overweightness and obesity found in this study among lesbian and bisexual females. For example, prior research points to the objectification theory, which suggests that sexual objectification experiences may, in part, explain eating disorder behavior [46]. Other contributing factors that have been suggested include discrimination [47] and a low concern for physical appearances [48].

This study had some limitations. BMI was calculated based on self-reported information provided by the survey respondents and may, therefore, be subject to response error. Another limitation is the cross-sectional nature of the study, which does not allow for identifying temporality. This study further strengthened the findings that there are differences between LGB subgroups’ associations with overweightness and obesity. Given the public health burden of overweightness and obesity, and the link with significant health consequences, this study contributed important information toward this issue that can be used for subpopulation prevention targeting.

## 5. Conclusions

Our results strengthen previous findings that lesbian and bisexual females have higher odds of being overweight or obese, whereas gay males have lower odds of being overweight or obese when compared to their straight counterparts. These findings further highlight the need for health research by sexual minority subgroup. Our rigorous analysis, which used a nationally representative sample and a PSM approach, contributes to the limited extant literature on overweightness and obesity among sexual minorities.

## Figures and Tables

**Table 1:** Weighted summary statistics of all study cohort, 2014–2017 (*n* = 716,609).

Characteristics	Straight	Lesbian or Gay	Bisexual	Other/ Don’t Know /Not Sure	*p*-Value
*n* (%)	685,096 (95.6)	10,353 (1.4)	11,255 (1.6)	9905 (1.4)	
Age					<0.0001 *
18–24	11.3	19.1	34.3	17.4	
25–34	15.5	20.8	27.2	18.1	
35–44	16.1	14.7	13.4	16.1	
45–54	17.8	20.5	9.8	12.9	
55–64	17.9	14.6	7.9	14.2	
65+	21.4	10.2	7.3	21.4	
Sex					<0.0001 *
Male	48.5	60.9	35.4	41.6	
Female	51.5	39.1	64.6	58.4	
Race					<0.0001 *
White	65.6	63.9	61.2	41.2	
Black	11.5	11.8	12.5	11.2	
Hispanic	14.9	15.1	15.5	36.0	
Others	8.0	9.3	10.7	11.6	
Education level					<0.0001 *
Did not graduate High School	12.9	7.7	14.1	36.1	
Graduated High School	28.7	23.4	28.5	29.5	
Attended College or Technical School	31.3	32.8	35.9	21.9	
Graduated from College or Technical School	27.1	36.1	21.5	12.4	
Employment					<0.0001 *
Yes	57.2	62.5	55.0	45.3	
No	42.8	37.5	45.0	54.7	
Region					<0.0001 *
Midwest	26.9	21.8	27.9	25.1	
Northeast	23.4	25.8	27.2	28.5	
South	31.7	31.1	28.9	28.4	
West	17.9	21.3	16.1	18.0	

*n* is unweighted frequency. * For categorical variables, weighted frequency are presented and *p* < 0.05 is considered significant.

**Table 2:** Pre- and post-propensity weighted means for covariates and absolute standardized mean difference (ASMD).

Covariates	Propensity Score Mean (Unweighted)				Propensity Score Mean (Weighted)			
	(ASMD from Straight)				(ASMD from Straight)			
	Straight	Gay or Lesbian	Bisexual	Other/Don’*t* Know/Not Sure	Straight	Gay or Lesbian	Bisexual	Other/Don’*t* Know/Not Sure
Age	0.36	0.20 (0.37)	0.17 (0.43)	0.47 (0.26)	0.35	0.34 (0.02)	0.35 (0.01)	0.37 (0.03)
Sex	0.57	0.58 (0.31)	0.64 (0.14)	0.36 (0.14)	0.43	0.44 (0.02)	0.44 (0.02)	0.42 (0.02)
Race	0.01	0.01 (0.01)	0.02 (0.02)	0.05 (0.20)	0.02	0.02 (0.01)	0.02 (0.01)	0.02 (0.01)
Education level	0.38	0.51 (0.29)	0.35 (0.05)	0.14 (0.50)	0.38	0.39 (0.03)	0.39 (0.03)	0.37 (0.02)
Employment status	0.50	0.59 (0.19)	0.53 (0.07)	0.32 (0.36)	0.50	0.50 (0.01)	0.49 (0.01)	0.47 (0.06)
Region	0.33	0.27 (0.14)	0.30 (0.06)	0.30 (0.07)	0.33	0.33 (0.01)	0.33 (0.01)	0.33 (0.01)

ASMDs are between straight and other sexual identity groups.

**Table 3:** Logistic regression of being at least overweight (BMI ≥ 25) on sexual identity status and by sex.

Sexual Identity Status	Full Sample	Male	Female
Lesbian or gay	0.93 (0.86, 1.01)	0.66 (0.59, 0.73) *	1.33 (1.17, 1.53) *
Bisexual	1.08 (1.00, 1.17) *	0.98 (0.86, 1.13)	1.21 (1.10, 1.34) *
Other/don’t know/not Sure	0.83 (0.73, 0.94) *	0.76 (0.62, 0.94) *	0.92 (0.79, 1.07)
Straight	Ref	Ref	Ref

* The odd ratios presented are significant. Ref = reference category and odds ratio and its 95% confident interval are presented in the Table.

**Table 4:** Logistic regression of obesity (BMI ≥ 30) on sexual identity status and by sex.

Sexual Identity Status	Full Sample	Male	Female
Lesbian or gay	1.02 (0.94, 1.11)	0.77 (0.69, 0.86) *	1.49 (1.31, 1.70) *
Bisexual	1.29 (1.18, 1.42) *	1.15 (0.98, 1.35)	1.43 (1.29, 1.59) *
Other/Don’t know/Not Sure	0.88 (0.78, 1.00) *	0.84 (0.68, 1.05)	0.92 (0.79, 1.06)
Straight	ref	ref	ref

* The odds ratios presented are significant. Ref = reference category and odds ratio and its 95% confident interval are presented in the Table.
